# Use of a Managed Solitary Bee to Pollinate Almonds: Population Sustainability and Increased Fruit Set

**DOI:** 10.3390/insects12010056

**Published:** 2021-01-11

**Authors:** Jordi Bosch, Sergio Osorio-Canadas, Fabio Sgolastra, Narcís Vicens

**Affiliations:** 1 Centre for Research on Ecology and Forestry Application, 08193 Bellaterra, Spain; 2 Departamento de Ecología de la Biodiversidad, Instituto de Ecología, Universidad Nacional Autónoma de México, AP 70-275, 04510 Mexico City, Mexico; s.osorio.canadas@gmail.com; 3 Dipartimento di Scienze e Tecnologie Agro-Alimentari, Alma Mater Studiorum Università di Bologna, Viale Fanin 42, 40127 Bologna, Italy; 4 Diputació de Girona, Servei de Medi Ambient, 17004 Girona, Spain; nvicens@ddgi.cat

**Keywords:** pollination service, *Osmia cornuta*, *Apis mellifera*, population dynamics, managed pollinators, crop pollination

## Abstract

**Simple Summary:**

Methods to rear *Osmia* bees to pollinate fruit trees have been developed in various parts of the world. These bees are excellent pollinators but evidence that their populations can be sustained in orchards and their use results in increased fruit production is scarce. We released an *Osmia cornuta* population at one end of an almond orchard. Then, we surveyed the pollinators visiting the almond flowers and measured fruit set in trees located at increasing distances from the nesting stations. We found that fruit production was higher in the trees that received more *Osmia* visits. Importantly, this result was obtained against a strong background of honeybees, which were 10 times more abundant than *Osmia*. The *Osmia* population obtained at the end of the flowering period was 1.28 larger than the population initially released. Our study demonstrates that *Osmia* populations can be sustained in orchard environments and that even a small population of a highly effective pollinator may have a significant impact on fruit set. Our results are encouraging for the use of *Osmia* populations and for the implementation of measures to promote wild pollinators in agricultural environments.

**Abstract:**

*Osmia* spp. are excellent orchard pollinators but evidence that their populations can be sustained in orchard environments and their use results in increased fruit production is scarce. We released an *Osmia cornuta* population in an almond orchard and measured its population dynamics, as well as visitation rates and fruit set at increasing distances from the nesting stations. Honeybees were 10 times more abundant than *O. cornuta*. However, the best models relating fruit set and bee visitation included only *O. cornuta* visitation, which explained 41% and 40% of the initial and final fruit set. Distance from the nesting stations explained 27.7% and 22.1% of the variability in initial and final fruit set. Of the 198 females released, 99 (54.4%) established and produced an average of 9.15 cells. Female population growth was 1.28. By comparing our results with those of previous *O. cornuta* studies we identify two important populational bottlenecks (female establishment and male-biased progeny sex ratios). Our study demonstrates that even a small population of a highly effective pollinator may have a significant impact on fruit set. Our results are encouraging for the use of *Osmia* managed populations and for the implementation of measures to promote wild pollinators in agricultural environments.

## 1. Introduction

Approximately three-quarters of the world’s crops benefit from animal pollination [1], and a significant part of this pollination service is provided by wild pollinators [2,3,4,5]. However, the current context of agricultural intensification, characterized by low crop diversity, increased crop field size, loss of semi-natural habitats, and increased pesticide use is clearly detrimental to pollinator abundance and diversity [6,7,8,9]. As a result, wild pollinators are notoriously scarce in many agricultural landscapes [10,11,12]. For this reason, the general perception is that wild pollinator populations are insufficient to provide adequate levels of pollination in intensively farmed areas, and populations of managed pollinators are usually introduced to enhance pollination services.

Fruit trees are highly dependent on pollinator visitation because they bloom for a short period of time in spring when weather conditions are often suboptimal for insect activity and because most cultivars are self-incompatible. For this reason, honey bee hives are usually introduced in orchards at a rate of 2–6 hives per ha, with each hive containing several thousands of foragers [13,14,15]. However, honey bees are only fully active at temperatures above 12–14 °C [16]. In addition, because they have long foraging ranges [17,18,19], and are highly generalist foragers, they often visit other flower species [14]. Finally, honey bees are not very effective fruit tree pollinators, mainly due to their low visit legitimacy (many of the visits result in no contact between the bee and the stigmas; [20,21,22,23,24,25]). These shortcomings, along with the risks associated with relying on a single species, have prompted the search for alternative pollinators, and methods to manage various *Osmia* species as orchard pollinators have been developed in different parts of the world [26,27]. These *Osmia* species are only active for a couple of months in spring and fly under marginal weather conditions [16]. In addition, they have short foraging ranges [28,29,30] and show a strong preference for fruit tree pollen [21,23,31,32,33,34,35]. Finally, *Osmia* visit legitimacy on fruit three flowers is close to 100%, and fruit set in flowers receiving a single visit is similar to fruit set in hand-pollinated flowers [20,21,23,24,25,36]. For these reasons, population densities recommended for orchard pollination with *Osmia* spp. are as low as 1250–2000 bees per ha (with a 1.6 male/female ratio) [26]. However, even if the pollinating effectiveness of *Osmia* spp. has been amply documented, evidence that the use of *Osmia* populations results in increased fruit production is still scarce [11,23,37,38].

The use of a managed pollinator is only sustainable if population levels can be maintained from year to year. Ideally, populations should be able to grow on site during the flowering period of the target crop. If this is not possible, population losses can be compensated by rearing populations under artificial conditions, as done with bumblebee colonies used for crop pollination [39]. However, attempts to mass-rear *Osmia* populations under artificial conditions have not been successful [40,41]. Therefore, to ensure the sustainability of *Osmia* spp. as managed pollinators it is essential to understand the dynamics of populations introduced in orchards. There are various factors potentially limiting the growth of *Osmia* populations released in orchards. These factors include: (a) Winter mortality (some of the cocoons introduced in the orchard contain individuals that are either dead or too weak to emerge); (b) low female establishment (some of the females that have successfully emerged out of their cocoon may be predated, too weak to start nesting activities, or disperse and nest away from the release site); (c) low fecundity (nesting females provision a low number of cells and therefore lay few eggs); (d) progeny mortality (a part of the progeny does not reach the adult stage due to either developmental failure or parasitism); (e) male-biased progeny sex ratio (nesting females produce a high proportion of males, which are less costly to produce than females [42]).

In this study, we released a population of the European species *Osmia cornuta* in an almond orchard. Almond pollination is particularly challenging because almonds bloom very early in the year (February-March) and have an unusually high bearing capacity (as many as 40% of the flowers may bear fruit) [43]. We measured female establishment and population growth, as well as flower visitation rates and fruit set at increasing distances from the nesting site. Our first objective was to assess whether the *O. cornuta* population had an impact on fruit production. Given the short foraging range of *O. cornuta*, we expected fruit set to be negatively correlated to distance from the nesting site. Our second objective was to establish whether the *O. cornuta* population could be increased on site and, by comparing our results with those of previous studies, to identify the main factors limiting population growth.

## 2. Materials and Methods

**Figure A1:**
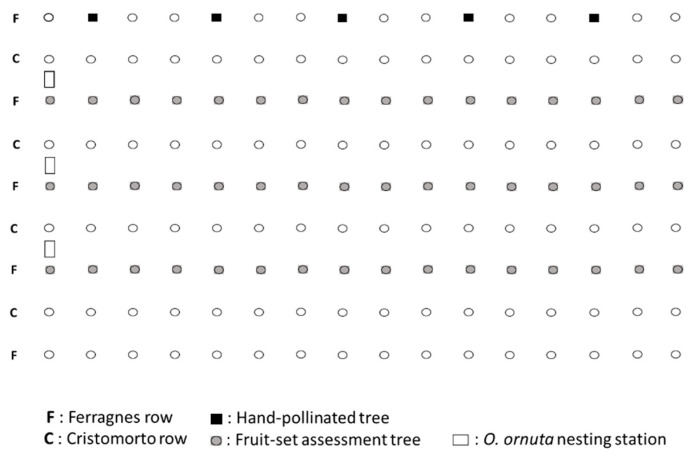
Structure of the study orchard and location of the *Osmia cornuta* nesting stations.

The study was conducted in an almond orchard in Vila-Seca (Tarragona, NE Spain). The orchard measured 0.5 ha and had 5 rows of the main cultivar Ferragnes intermixed with 4 rows of the pollinizer cultivar Cristomorto (both cultivars are self-incompatible) (Figure A1). Each row had 16 trees. The orchard was located within a matrix of farmland, including cereal fields, fallow land, and a mixture of olive, carob, and hazelnut orchards. There were no honey bee hives in the orchard or its surroundings (at least within a 500 m radius).

In early February 1992, prior to almond bloom, we set up 3 nesting stations for *O. cornuta* at one edge of the orchard (Figure A1). Nesting stations were made with wooden boxes with the front side open held 1.5 m above the ground on four metal fence posts. Each station contained 15 perforated solid wood blocks with 25 inserted paper straws (length: 15 cm; inside diameter: 8 mm). On 22 February, before the orchard started to bloom, we placed 198 females and 360 males (M/F sex ratio: 1.8) within their cocoons in open cardboard boxes inside the nesting stations. These cocoons had been wintered at 4 °C since October 1991.

To determine the number of females that established at the nesting stations, we inspected each nesting cavity with an otoscope. This was done at night, when females were roosting inside their nests, seven times during the nesting period (approximately twice a week).

During peak bloom, we conducted pollinator counts on the central row of Ferragnes. An observer slowly walked around each tree and noted all pollinators seen visiting the flowers. Total observation time was 10 h and 40 min (40 min × 16 trees).

To assess fruit set (% of flowers that set fruit) at increasing distances (0–80 m) from the *O. cornuta* nesting shelters, we marked all trees in 3 Ferragnes rows (48 trees), including the row used in the pollinator counts (Figure A1). Before bloom we tagged 4 branches on each tree and counted the number of flower buds on each branch (mean ± SE = 264.4 ± 1.6 flower buds per branch). In April, we counted the number of initiated fruits on each branch (initial fruit set). Following the natural fruit drop, fruits were counted again in June when they had reached their final size (final fruit set).

To obtain a measure of maximum potential fruit set, we marked 5 additional Ferragnes trees (Figure A1) on which we tagged 4 branches and counted flower buds as described above. These trees were checked daily and newly opened flowers were hand-pollinated with Cristomorto pollen. Initial and final fruit set was assessed as described above.

After petal fall, when *O. cornuta* nesting activity had ceased, we removed nesting materials and kept them in the laboratory. In September, when development was completed, we pulled out paper straws and analyzed the contents of each nest. We quantified the number of male and female offspring produced (female cocoons are typically larger than male cocoons and are found in the inner cells of the nest [42]). We also recorded offspring mortality.

### Statistical Analysis

Initial and final fruit set were analyzed separately. To analyze whether fruit set declined with distance from the *O. cornuta* nesting stations, we used Linear Mixed Models (LMMs) with a tree as a random factor. We considered a linear relationship (model with distance as the only explicative variable) and a quadratic relationship (model with distance and distance^2^). We then used a model inference approach [44] to select the best-fit model based on AICc values using maximum likelihood criteria. Models with ΔAICc < 2 were considered equal to the best model [44]. We then run a LMM model with REML to obtain unbiased parameter estimates. We calculated a likelihood-ratio-based R^2^ of the best models as a measure of explanatory power.

To analyze whether fruit set was related to *O. cornuta* and/or *A. mellifera* visitation, we conducted a model selection procedure with LM models, testing all possible explanatory variable combinations through a multi-model inference approach with the ‘dredge’ function (‘MuMin’ package, [45]). We again selected the best models based on AICc values. Following model selection, we used a model averaging procedure (with averaged variable coefficients) based on AICc. This was done with the ‘model.avg’ function (‘MunMin’ package), which yields model-averaged estimates of variable coefficients and p-values for each explanatory variable. Conditional average and full average approaches yielded almost identical results. We show only conditional average results. Finally, we calculated the adjusted-R^2^ of the best models (containing significant explanatory variables) as a measure of explanatory power.

Percent initial and final fruit set were arcsin-transformed. The distribution of residuals was visually inspected for homoscedasticity and the normality assumption was tested with the Shapiro test. All analyses were conducted with the ‘nlme’ [46] and MuMin packages in R [47]. All means are followed by standard error (SE).

## 3. Results

Maximum temperatures during the days following the release of the *O. cornuta* population (22 February) ranged between 14 and 18 °C. The first females engaged in nesting activities were observed on 28 February. Of the 198 females released, 182 emerged out of their cocoons (91.9 % winter survival). The maximum number of females established in the nesting stations was counted on March 3 (99 females; 54.4% of the emerged females).

**Figure 1:**
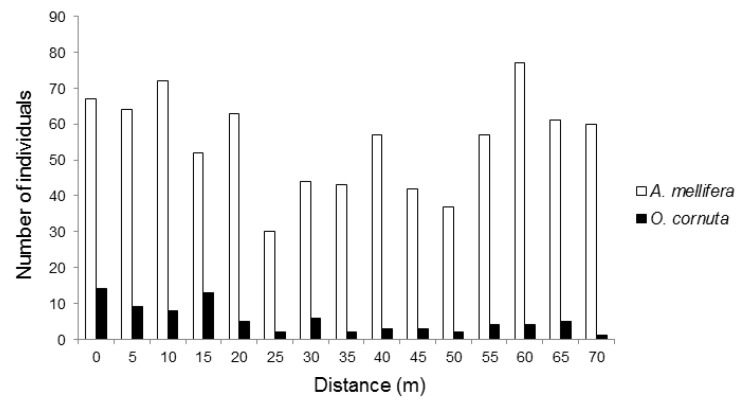
Number *Apis mellifera* and *Osmia cornuta* individuals recorded visiting almond flowers at increasing distances from the *O. cornuta* nesting stations.

We recorded 1114 pollinators visiting the almond flowers. Although there were no hives in sight, the most frequent pollinator was, by far, *Apis mellifera* (74.1% of the visits recorded), followed by *O. cornuta* (7.3%). Other visitors included various flies (16.2%), hoverflies (4.11%, *Eristalis tenax*, *Eupeodes* sp.), and wild bees (1.1%, *Andrena nigroaenea*, *Andrena* sp., *Eucera* sp., *Bombus terrestris*, *Xylocopa violacea*). Most (70.4%) *O. cornuta* visitation occurred within 30 m from the nesting stations (Figure 1). *Apis mellifera* visitation followed an irregular pattern across the orchard and tended to be higher towards the two ends of the orchard (Figure 1).

Almond bloom was over by 23 March. At that time, *O. cornuta* females that were still alive foraged mostly on *Diplotaxis erucoides* (Brassicaceae), a common weed in the surroundings of the orchard. By 29 March *O. cornuta* nesting activity had ceased. The number of nests produced was 203. These nests contained 253 female and 653 male cells. Offspring mortality was 7.1%. Most of this mortality was due to unknown causes (5.3%), and the rest to parasitism by the cleptoparasitic mite *Chaetodactylus osmiae* (1.8%). The live female population recovered was 241, and the live male population 601 (M/F sex ratio: 2.5).

**Figure 2:**
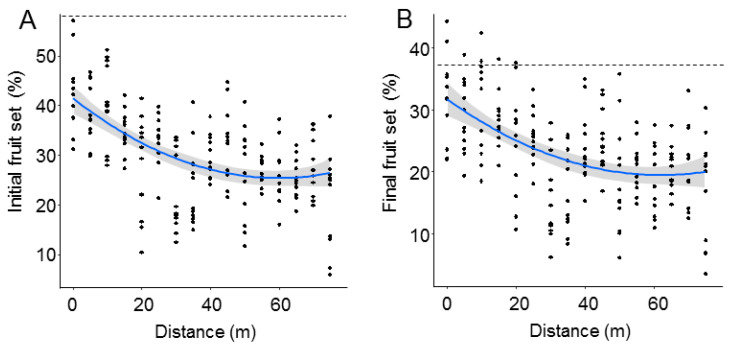
Initial (**A**) and final (**B**) fruit set (% of flowers setting fruit in April and June, respectively) at increasing distances from the *Osmia cornuta* nesting shelters. Each dot represents a branch. Broken lines indicate mean fruit set in hand-pollinated trees (five trees; four branches per tree). The gray bands represent 95% confidence intervals.

**Figure 3:**
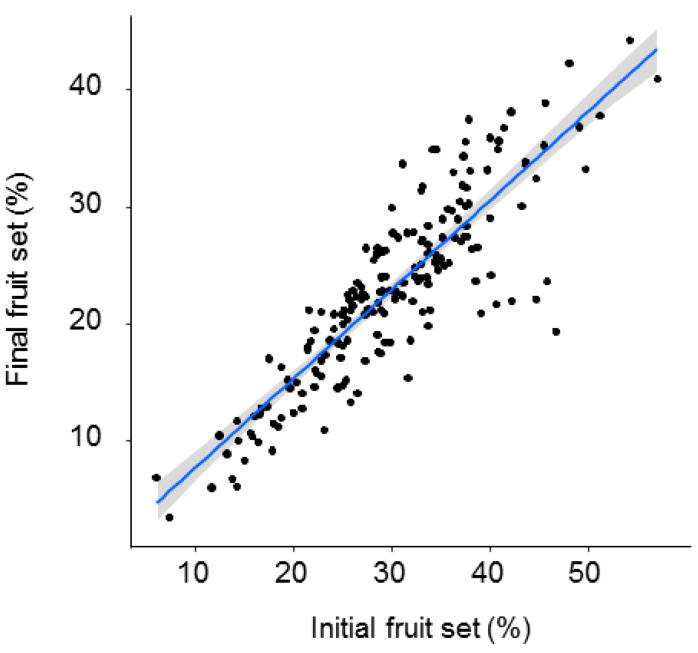
Relationship between initial (April) and final (June) fruit set (% of flowers setting fruit). Each dot represents a branch. The gray band represents the 95% confidence interval.

**Figure A2:**
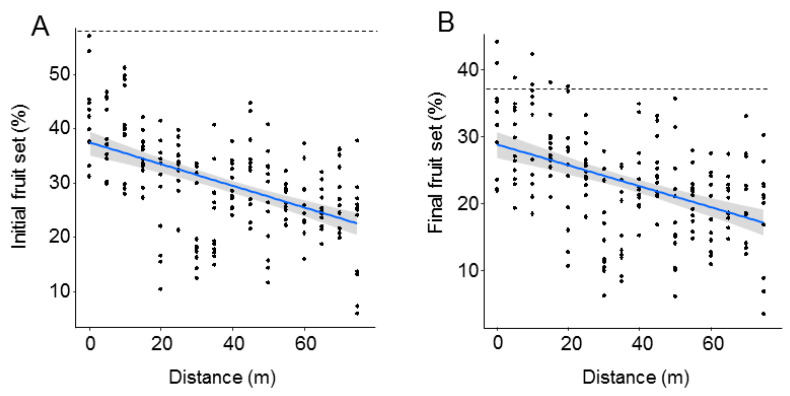
Initial (**A**) and final (**B**) fruit set (% of flowers setting fruit in April and June, respectively) at increasing distances from the *Osmia cornuta* nesting shelters. Each dot represents a branch. Broken lines indicate mean fruit set in hand-pollinated trees (5 trees; 4 branches per tree). The gray bands represent 95% confidence intervals.

Both initial and final fruit set significantly declined with distance from the *O. cornuta* nesting stations (Figure 2). The quadratic model provided the best fit, with distance explaining 27.7% of the variability in initial fruit set and 22.1% of the variability in final fruit set (Table 1). Importantly, the random factor tree also had a strong effect on fruit set (initial: 38.7%; final: 37.1%). The linear models yielded similar results (Table A1 and Figure A2 in Appendix A). Final fruit set was strongly related to initial fruit set (Pearson’s r = 0.88; *p* < 0.0001; Figure 3).

**Figure 4:**
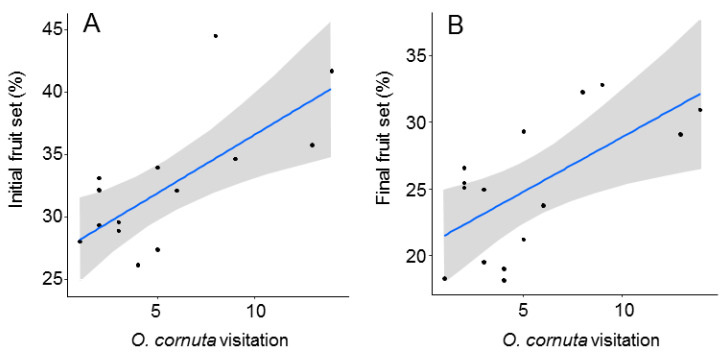
Relationship between *Osmia cornuta* visitation and initial (**A**) and final (**B**) fruit set (% of flowers setting fruit in April and June, respectively). Each dot represents a tree. Gray bands represent 95% confidence intervals.

The best models analyzing the effect of *O. cornuta* and *A. mellifera* visitation on fruit set included only *O. cornuta* visitation, which explained 41% of the initial fruit set and 40% of the final fruit set (Table 2; Figure 4). Even then, the levels of fruit set obtained across the orchard were lower than those obtained in the five hand-pollinated trees (initial fruit set: 57.8 ± 2.0; final fruit set: 36.9 ± 1.8%) (Figure 2), indicating that pollination services could still be increased.

## 4. Discussion

Our first objective was to assess whether the *O. cornuta* population had an impact on almond production. Most of the *O. cornuta* observed were recorded within 30 m from the nesting stations. Although *Osmia* females are able to locate their nest from distances as far as 500–1800 m [28,29], populations established in orchards concentrate most of their foraging within 50 m of the nesting sites [23,30]. The negative relationship between fruit set and distance from the nesting stations closely paralleled the distribution of *O. cornuta* across the orchard. The contribution of *O. cornuta* to pollination service was further confirmed by the analysis identifying *O. cornuta* visitation (but not *A. mellifera* visitation) as a significant predictor of initial and final fruit set. This result is remarkable given that *A. mellifera* visitation was 10 times higher than *O. cornuta* visitation. *Apis mellifera* is consistently reported as the dominant pollinator species in commercial orchards (e.g., [11,12,48]). However, its contribution to pollination service is strongly limited by its low per-visit pollination effectiveness on fruit tree flowers [20,21,22,23,24,25]. Our results are limited to a single orchard and a single year. However, they are in line with a previous study that found a significant impact of *O. cornuta* visitation on final seed-set in a pear orchard in which *A. mellifera* was 7 times more abundant [23]. These results are also in agreement with studies showing yield increases in orchards pollinated with other *Osmia* species [11,37,38]. Our study also shows that initial fruit set (commonly used as a proxy of pollination service) is a good predictor of final fruit set in almonds.

Our second objective was to establish whether the *O. cornuta* population could be increased on site, and to identify the main factors limiting population growth. We released 198 females of which 182 emerged out of their cocoons (8.1% winter mortality). This winter mortality is similar to values obtained in other *O. cornuta* populations managed for orchard pollination (Table 3). Of the 182 females that emerged, 99 (54.4%) established in the nesting stations. Failure to establish can be caused by lack of vigor of emerging females [49], predation [50,51,52], and dispersal of pre-nesting females [53], but attributing a relative weight to each of these three factors is not easy. At any rate, the percent establishment obtained in our study was similar to values obtained with other *O. cornuta* populations released in orchards (Table 3). The 99 females that established in the nesting stations produced 906 cells (an average of 9.15 cells/female). This fecundity is again close to fecundity in other *O. cornuta* populations released in orchards (Table 3). Of these 906 cells, 253 contained female progeny (2.6 M/F sex ratio). This is considerably higher than that the sex ratio of the population released (1.8), but similar to sex ratios obtained from other *O. cornuta* populations released in orchards (Table 3). Developmental mortality (5.3%) and parasitism (1.8%) were also similar to mortality levels obtained in previous studies (Table 3). Ultimately, the female population recovered was 1.28 times larger than the female population released, providing evidence that *Osmia* populations can be sustained in orchard environments ([26] and references therein, [54]).

The results of Table 3 allow us to identify two important bottlenecks in the dynamics of *O. cornuta* populations managed for orchard pollination. The first one occurs in the establishment phase, during which the effective female population is reduced by ca. 50%. Previous studies have shown that *Osmia* establishment can be enhanced by releasing populations within their natal nests, rather than as loose cocoons [33,55,59,60,61]. This result is probably mediated by olfactory nest cues that enhance the tendency of females to re-nest in their natal nesting site (philopatry; [61]). In relation to this, an olfactory attractant similar to that developed for *Osmia lignaria* [54] could potentially enhance establishment of *O. cornuta* populations released in orchards. The second important bottleneck is the production of a male-biased sex ratio. In *O. cornuta*, the sex ratio of populations trap-nested in semi-natural areas is 1.5 ± 0.06 (*n* = 4 populations) [62,63], a figure that closely matches the theoretically optimal sex ratio based on male and female body weights (1.7) [42]. However, progeny sex ratios obtained from populations released in orchards are consistently higher (ca. 2.5; Table 3). In other words, in populations nesting in orchards, a considerable fraction of the parental investment is devoted to the production of surplus males. The causes underlying these differences in sexual allocation are unclear, but a greater proportion of female cells in managed populations could be obtained by increasing the diameter [56,64,65], or the length [55,65] of the nesting cavities offered in orchard operations. A more balanced sex ratio (closer to 1.7) would increase the reproductive and pollinating potential of populations recovered from orchards.

## 5. Conclusions

Our study demonstrates that even a small population of a highly effective pollinator may have a significant impact on crop pollination service and fruit set. Our results are encouraging not only for the use of *Osmia* spp. as managed pollinators but also for the implementation of measures to protect wild pollinator communities in orchard environments. In addition to *Osmia* spp., various species of *Andrena*, *Eucera* and *Bombus* are highly effective fruit tree pollinators [5,22,24,25,36]. In the current scenario of pollinator declines, agri-environmental measures to enhance wild populations of these valuable pollinators could have important economic returns in terms of enhanced pollination service and fruit yields.

## Figures and Tables

**Table 1:** Output of Linear Mixed Model (LMM) relating initial fruit (A) and final fruit set (B) to distance from the *Osmia cornuta* nesting shelters. Parameter- (*t*) and *p*-values are provided for the best-fitted models based on AICc selection. R^2^m and R^2^c are the marginal and conditional R^2^ of the best-fitted models.

	A. Initial Fruit Set		B. Final Fruit Set	
	*t*-Value	*p*-Value	*t*-Value	*p*-Value
(Intercept)	25.27	<0.0001	22.63	<0.0001
Distance	−3.33	<0.0001	−2.85	0.0001
Distance^2^	2.12	0.0395	1.75	0.0857
	R^2^m: 0.28; R^2^c: 0.67		R^2^m: 0.22; R^2^c: 0.59	

**Table 2:** Output of LM model averaging relating initial (A) and final (B) fruit set to *Osmia cornuta* and *Apis mellifera* visitation. Estimated coefficients and their *p*-values are provided. Adjusted R^2^ of the best-fitted model (containing only *O. cornuta* visitation as predictor variable) are provided.

(A) Initial Fruit Set.					
	Estimate	SE	Adjusted SE	*z* Value	Pr(>|z|)
(Intercept)	0.5545	0.028	0.031	17.62	<2e−16
*O. cornuta*	0.0099	0.003	0.003	3.08	0.002
*A. mellifera*	−0.0004	0.001	0.001	0.39	0.689
Adjusted R^2^: 0.44					
**(B) Final Fruit Set**					
	**Estimate**	**SE**	**Adjusted SE**	***z* Value**	**Pr(>|z|)**
(Intercept)	0.489	0.043	0.046	10.63	<2e−16
*O. cornuta*	0.009	0.003	0.003	2.70	0.007
*A. mellifera*	−0.001	0.001	0.001	1.17	0.241
Adjusted R^2^: 0.36					

**Table 3:** Population parameters of *Osmia cornuta* populations released in orchards. All means followed by standard error (SE).

	This Study	Other Studies ^7^
Winter mortality ^1^	8.1%	5.5 ± 0.7 (*n* = 8)
Female establishment ^2^	54.4%	50.7 ± 5.1 (*n* = 14)
Fecundity ^3^	9.2	9.8 ± 0.9 (*n* = 10)
Progeny sex ratio ^4^	2.6	2.5 ± 0.18 (*n* = 12)
Developmental mortality ^5^	5.3%	8.5 ± 0.9 (*n* = 15)
Parasitism ^6^	1.8%	1.6 ± 0.4 (*n* = 17)

^1^ Individuals that did not emerge out of their cocoons; ^2^ Proportion of emerged females that established at the nesting stations provided; ^3^ Number of eggs laid; ^4^ Males/females; ^5^ Proportion of progeny that died from unknown causes; ^6^ Proportion of progeny that died from cleptoparasitism; ^7^ [21,23,53,55,56,57,58].

## Data Availability

Data are available in the Supplementary Materials.

## Supplementary Materials

The following are available online at https://www.mdpi.com/2075-4450/12/1/56/s1, dataset. Table S1: Fruit set vs. Distance; Table S2: Osmia vs. fset row1.

## Appendix A

**Table A1:** Output of Linear Mixed Model (LMM) relating initial fruit (A) and final fruit set (B) to distance from the *Osmia cornuta* nesting stations considering a linear fit. R^2^m and R^2^c are the marginal and conditional R^2^ of the best-fitted models.

(A) Initial Fruit Set		
	*t*-Value	*p*-Value
(Intercept)	32.03	<0.0001
Distance	−4.73	<0.0001
R^2^m: 0.24; R^2^c: 0.66		
**(B) Final Fruit Set**		
	***t*-Value**	***p*-Value**
(Intercept)	29.22	<0.0001
Distance	−4.31	0.0001
R^2^m: 0.19; R^2^c: 0.59		
